# FUNDAMENTAL MEASUREMENTS ON OPTICALLY PREPARED ATOMS: A WORKSHOP

**DOI:** 10.6028/jres.092.014

**Published:** 1987-04-01

**Authors:** Robert E. Drullinger

**Affiliations:** Workshop Chairman, Time and Frequency Division, Center for Basic Standards, National Bureau of Standards, Boulder, CO 80303

Measurements of our fundamental scaling parameters (e.g., R_∞_, α, m_e_/m_p_) as well as tests of our basic theories of matter and radiation are frequently obtained from high resolution spectroscopic measurements on atomic systems. The new and evolving technologies of laser spectroscopy, laser cooling and trapping of atoms as well as squeezed states will have a dramatic impact on this area of metrology. While these topics have received a great deal of attention in their own right, there has not been a forum in which their impact on the field of fundamental measurements has been specifically addressed. For this reason, the National Bureau of Standards hosted a workshop on “Fundamental Measurements on Optically Prepared Atoms” on September 29–30, 1986.

The workshop was divided into four half-day sessions. The first dealt with atomic states and structural parameters. It was highlighted by talks involving the quantum mechanical aspects of the atom-photon interaction. The exchange of photons between atoms and the light field were treated in a way that illustrated phenomena such as anti-bunching, squeezed states and optical bistability. Similarly, the interaction of a single atom with several resonant fields was even shown to allow observation of the internal state of the atom in real time (observation of “quantum jumps”). The second session dealt mainly with the kinetic state or velocity of atoms. Demonstration of highly efficient new laser-cooling techniques based on stimulated processes as well as spontaneous emission and speculation on what may be possible with ultra-cold atoms made for a very lively and exciting session. The idea that an atom placed in a box and cooled to a temperature so low that the deBroglie wavelength is long compared to atomic dimensions might lead to confinement with almost no perturbation from encounters with the walls. If verified experimentally, this idea could have profound consequences for precision measurements.

The third session dealt with the limits to measurement accuracy as we understand them today and how those limits come into play with the new spectroscopic and cooling techniques. The last session dealt with an important applied field: the use of optically prepared atoms in frequency standards. Since frequency standards have many orders of magnitude more precision and accuracy than any other standard, they represent a great testing ground for the concepts discussed during the workshop.

The workshop was attended by 45 people. Seven countries were represented with 40% of the attendees from outside the United States. Twenty-seven papers were presented in the four half-day sessions, which had to be augmented with an evening session to accommodate the lively discussion that followed most of the papers. The format of informal talks with no subsequent publication of papers was designed to encourage speculation and judging from the discussion during and following many of the talks, we were quite successful.

A list of the workshops and talks follow:

## Internal States of Atoms

H. J. Kimble:Non-Classical Dynamics With Intra-Cavity AtomsP. Toschek:Absorption by the Numbers: Recent Spectroscopy of Trapped IonsD. McIntyre:Two-Photon Optical Ramsey Spectroscopy of Freely Falling AtomsL. Hunter:Search for an Electric Dipole Moment of the ElectronT. Bergeman:Proposed Application of Decelerated Atomic Beams to Observe Long-Lived (Interference Stabilized) Stark ResonancesS. A. Lee:Fast Beam Laser Spectroscopy: Present and FutureW. Fairbank:Precision Wavelength Measurement of Te_2_ Reference Lines Near Hydrogen and Positronium Transitions at 4880Å

## External Atomic States

S. Chu:Laser Cooling and Trapping of Atoms: Where are the Limits?C. Salomon:Cooling Atoms With Stimulated EmissionJ. Hall:Some Ideas About Experiments With Freely Falling AtomsW. Ertmer:Preparation of Cold Atoms for Precision MeasurementsW. Phillips:New and Future Experiments on Cooling and Trapping of Neutral AtomsF. Plumelle:Experiments on Laser Cooled Mg^+^ IonsB. Jaduszliwer:Electron-Cesium Collisions With Optical State Preparation and AnalysisJ. Bahns:On Containerless Condensation of “Mirror” Matter

## Measurement Limitations and Systematic Effects

D. Wineland:Fundamental Limits to Spectroscopic AccuracyJ. S. Boulanger:Requirements for Evaluatable EnvironmentsJ. Shirley:Majorana Effects in Atomic BeamsA. DeMarchi:Does Spin Exchange Limit the Density of Neutral Vapors for Accurate Measurements?G. Hanes:Candidate Ions for Extended Observation PeriodsR. Douglas:Multiphoton Ionization for Atom-State Detection or State Preparation

## Optically Pumped Frequency Standards

G. Theobald:Detailed Studies of Cesium Beam Optical Pumping; Applications to an Atomic ClockJ.-L. Picqué:Laser Cooling and Optically Pumped Cesium Beam Frequency StandardsA. Clairon:The LPTF Optically Pumped Cesium Frequency StandardR. Drullinger:Design of the NBS Optically Pumped Frequency StandardM. Ohtsu:Ultrahigh Sensitive Frequency Discrimination in Diode Laser Pumped ^87^Rb Atomic ClocksH. Robinson:The Temperature Dependence of the Wall Shift in Some Evacuated Rb Cells

